# Attitudes and opinions regarding confirmatory adaptive clinical trials: a mixed methods analysis from the Adaptive Designs Accelerating Promising Trials into Treatments (ADAPT-IT) project

**DOI:** 10.1186/s13063-016-1493-z

**Published:** 2016-07-29

**Authors:** William J. Meurer, Laurie Legocki, Samkeliso Mawocha, Shirley M. Frederiksen, Timothy C. Guetterman, William Barsan, Roger Lewis, Donald Berry, Michael Fetters

**Affiliations:** 1 Department of Emergency Medicine, University of Michigan, TC B1-354 1500 E. Medical Center Drive, Ann Arbor, MI 48109 USA; 2 Department of Neurology, University of Michigan, TC B1-354 1500 E. Medical Center Drive, Ann Arbor, MI 48109 USA; 3 Department of Family Medicine, University of Michigan, Ann Arbor, MI 48109 USA; 4 Harbor-UCLA Medical Center, Torrance, CA 90502 USA; 5 University of Texas MD Anderson Cancer Center, Houston, TX 77030 USA

**Keywords:** Adaptive clinical trials, Mixed methods research, Bayesian statistics, Emergency medicine, Neurology, Clinical trials

## Abstract

**Background:**

Adaptive designs have been increasingly used in the pharmaceutical and device industries, but adoption within the academic setting has been less widespread — particularly for confirmatory phase trials. We sought to understand perceptions about understanding, acceptability, and scientific validity of adaptive clinical trials (ACTs).

**Methods:**

We used a convergent mixed methods design using survey and mini-focus group data collection procedures to elucidate attitudes and opinions among “trial community” stakeholders regarding understanding, acceptability, efficiency, scientific validity, and speed of discovery with adaptive designs. Data were collected about various aspects of ACTs using self-administered surveys (paper or Web-based) with visual analog scales (VASs) with free text responses and with mini-focus groups of key stakeholders. Participants were recruited as part of an ongoing NIH/FDA-funded research project exploring the incorporation of ACTs into an existing NIH network that focuses on confirmatory phase clinical trials in neurological emergencies. “Trial community” representatives, namely, clinical investigators, biostatisticians, NIH officials, and FDA scientists involved in the planning of four clinical trials, were eligible to participate. In addition, recent and current members of a clinical trial-oriented NIH study section were also eligible.

**Results:**

A total of 76 stakeholders completed the survey (out of 91 who were offered it, response rate 84 %). While the VAS attitudinal data showed substantial variability across respondents about acceptability and understanding of ACTs by various constituencies, respondents perceived clinicians to be less likely to understand ACTs and that ACTs probably would increase the efficiency of discovery. Textual and focus group responses emerged into several themes that enhanced understanding of VAS attitudinal data including the following: acceptability of adaptive designs depends on constituency and situation; there is variable understanding of ACTs (limited among clinicians, perceived to be higher at FDA); views about the potential for efficiency depend on the situation and implementation. Participants also frequently mentioned a need for greater education within the academic community. Finally, the empiric, non-quantitative selection of treatments for phase III trials based on limited phase II trials was highlighted as an opportunity for improvement and a potential explanation for the high number of neutral confirmatory trials.

**Conclusions:**

These data show considerable variations in attitudes and beliefs about ACTs among trial community representatives. For adaptive trials to be fully considered when appropriate and for the research enterprise to realize the full potential of adaptive designs will likely require extensive experience and trust building within the trial community.

**Electronic supplementary material:**

The online version of this article (doi:10.1186/s13063-016-1493-z) contains supplementary material, which is available to authorized users.

## Background

Randomized controlled trials represent the gold standard for discovering new treatments for disease. Clinical trial designs have evolved over time, yet innovations in design have not commonly been observed in large-scale confirmatory trials. Recently, the National Institutes of Health (NIH) and the Food and Drug Administration (FDA) have taken interest in the potential of adaptive designs for accelerating the bench-to-bedside transition of new scientific breakthroughs [1].

Adaptive clinical trial (ACT) designs use accumulating data on patients from within a trial to make decisions about the future conduct of a trial [2, 3]. Adaptations can include comparisons of multiple dose tiers, response adaptive randomization (i.e., more subjects randomized to dose tiers with a greater probability of effect or a lower probability of toxicity), sample size re-estimation, and efficacy/futility stopping rules. In order to be able to define type I error rates, all potential changes to the trial must be pre-specified [4, 5]. Adaptive designs have been increasingly used in the pharmaceutical and device industries, but adoption within the academic setting has been less widespread — particularly for confirmatory phase trials [6–8].

As an emerging form of clinical trial with some features that seemingly contradict some perceptions about “traditional, fixed clinical trials,” ACTs may be perceived as a threat to the status quo [9]. Particularly in the age of team science, success of the research enterprise is dependent upon collaboration and communication against multiple constituencies [10]. An understanding of the opinions of the “trial community,” namely, clinical investigators, biostatisticians, NIH officials, FDA scientists, and study section members, could help define current attitudes and beliefs about adaptive designs and ACTs. These multiple constituencies are all key stakeholders in the clinical trial enterprise, and a better understanding of their attitudes and beliefs about acceptability and understanding of ACTs, efficiency, scientific validity, and speed of discovery could provide a window on the perceived place of ACTs in contemporary clinical science.

Currently, relatively little published data exists regarding the opinions of the academic trial community, particularly with respect to ACT use for confirmatory phase trials. Surveys of industry trial stakeholders, mostly from pharmaceutical developers in 2008 and 2012, found that managing the changes during trial operation was the largest barrier; education and time/resources for planning were minor concerns [11, 12]. Given this gap in the literature, here we aimed to describe the views of constituencies from academia and government participating in the Adaptive Designs Accelerating Trials into Treatments (ADAPT-IT) project, and compare their beliefs about understanding and acceptability of adaptive designs, and about efficiency, scientific validity, and speed of discovery with adaptive designs.

## Methods

### Study design

We conducted a convergent mixed methods design that used a 22-item ACT beliefs survey with categorized visual analog scales (VASs), free text survey responses, and mini-focus groups, a type of group interview that focuses on three or four respondents and helps facilitate the participation of all in the discussion versus a larger conventional focus group [13, 14]. Additionally, the mini-focus groups were employed to maintain homogeneity among the types of stakeholders (having the academic biostatisticians together) and to enhance the interactivity of the groups. A mixed methods approach was implemented to elucidate participants’ beliefs, to identify the reasoning behind the beliefs expressed, and to integrate the data to provide the broadest possible understanding. Study instruments were developed by experts in both traditional and adaptive clinical trials (WJM, RL) and a mixed methodologist and ethicist (MF) and focused on participants’ beliefs about ACT designs. The ADAPT-IT mixed methods evaluation team previously reported on the opinions regarding the ethics of ACT designs from data from a subset of respondents in the current report [15]. In addition, we published perspectives and lessons learned from the overall trial planning process employed by ADAPT-IT [16]. We structured our study design and reporting in accordance with the consolidated criteria for reporting qualitative research (COREQ) statement (see Additional file 1) [17].

### Settings and participants

Participants were recruited as part of an ongoing NIH/FDA-funded research project exploring the incorporation of ACT designs into an existing Neurological Emergencies Treatment Trials (NETT) Network [1, 18]. So far, one of the trials planned in ADAPT-IT has been fully planned, funded, and is enrolling patients, and there is another prospective comparison of a pre-specified adaptive design to an ongoing clinical trial that was developed; the other projects are still being developed [19, 20]. Clinicians, preclinical scientists, and biostatisticians who were planning clinical trials were initially recruited as part of the grant proposal for ADAPT-IT; after it was funded, all were invited to the trial planning meetings and survey. We intentionally sampled for key trial leaders for the mini-focus groups. Project investigators held a series of meetings that included experts in ACT design and investigators interested in developing an ACT for specific research topics related to neurological emergencies. A mixed methods team assessed the ACT development process during these meetings and conducted the analysis. Since several trials were developed over time with different teams, responses in the current investigation are from baseline surveys or mini-focus groups that occurred prior to any ADAPT-IT trial planning activities.

### Data collection

Data were collected with self-administered surveys, either by paper or on the Web, by using VAS and free text responses. The survey was revised after an initial pilot phase where it was administered to clinician researchers experienced in clinical trials and survey administration, but who were not otherwise involved in the project. Data also were collected during five mini-focus groups with four to six clinical trial experts per group [13, 14]. ADAPT-IT participants from clinical medicine and biostatistics who were present for trial planning meetings were recruited for the mini-focus groups. The mini-focus group guide was specifically designed with topics to parallel the items on the VAS instrument so that results from both instruments could be mapped together. Data were collected between January and August of 2011. Participants were classified as belonging to one of the following groups of clinical trial experts: academic biostatisticians from NIH-funded clinical trial networks (*n* = 5) with substantial experience running phase III trials, consultant biostatisticians working in academic or industry settings with specific experience in Bayesian adaptive designs (*n* = 6), academic clinician researchers (*n* = 22), and other stakeholders, e.g., NIH officials, FDA statisticians, medical officers, and patient advocates — all experts in the planning of clinical trials (*n* = 20). Recent and current members of a clinical trial oriented NIH study section (grant review panel) were also eligible (*n* = 23). No patients were surveyed for this report. Instead we asked those surveyed for their opinions about how patients might view the advantages and disadvantages of ACTs. These individuals were offered an anonymous Web-based version of the VAS survey. The survey questions were piloted and revised for clarity and purpose on clinical trial experts not otherwise involved in ADAPT-IT. We used the paper survey only at the first in-person trial planning meeting, and we transitioned to an identical, Web-based version for all subsequent data collection. The VAS survey and discussion guide are available at the following links from our previous publication focused on the ethical themes from this part of the study [15]:

- VAS survey: http://www.biomedcentral.com/content/supplementary/s12910-015-0022-z-s1.doc
- Discussion guide: http://www.biomedcentral.com/content/supplementary/s12910-015-0022-z-s2.doc

### Variables and responses

Survey and mini-focus group questions were formulated to gather opinions of the clinical trial experts regarding scientific, logistical, and other advantages and disadvantages of ACT designs. Participants considered advantages and disadvantages from the perspectives of the patient, the researcher, and society as a whole.

### Data sources

Mini-focus groups with clinical trial experts were conducted before the initial face-to-face scientific planning meetings for four of the five trials [13, 14]. These mini-focus group sessions were digitally recorded (audio only) and transcribed verbatim, and the data were entered into Atlas.ti v6.0 for data management, coding, and analysis [21].

Participants answered the VAS items by completing a paper survey or a Web-based survey. The VAS allowed participants to mark a point of agreement on a continuum ranging from “definitely not,” to “probably not,” to “possibly,” to “probably,” to “definitely.” We used a 100-point scale to allow more resolution to examine differences than a 5-point structured Likert scale would allow, as we desired respondents to make estimations on a probability scale. To compute a quantitative measure of a participant’s assessment, we assigned the lowest anchor a value of 0 and the highest anchor a value of 100 and calculated a level of agreement score based on the point chosen by the participants for the VAS items. The Web-based survey provided a number, so it was clear to respondents that the entire scale could be used.

### Data analysis

Descriptive statistics (proportions and means) were calculated for demographic variables. The VAS data were depicted by using box plots visually illustrating the median, interquartile range (IQR), and outliers (outside 1.5 times the IQR) for each survey question. Regarding the qualitative analysis, open coding was conducted initially, then reviewed together to develop a coding scheme; this was then used independently for several transcripts to refine interpretation of the codes. The five mini-focus groups and free text responses from the surveys were analyzed independently by two investigators (LL, SMF) from the mixed methods evaluation team with an 88 % intercoder agreement [22]. The development of a coding scheme was based initially on the thematic basis of the interview guide and revised to reflect the primary themes that emerged from the analysis. The VAS scores using box plot diagrams were integrated with comments by constituency groups for subanalyses to merge quantitative ratings with qualitative textual data and with representative quotations [23]. The attitudinal data from the VASs were organized to facilitate comparisons by the respondent constituencies, and key domains, understanding and acceptability of ACTs, efficiency, scientific validity, and speed of discovery.

We used the qualitative analytic procedures of immersion (researchers immersing themselves in the data) and crystallization (researchers reflecting on how ideas about what meanings in the data crystallize out as major findings). The coding scheme evolved to have seven major domains: ACTs-General Issues (four subthemes), Logistics (three subthemes), Ethics (four subthemes), Regulatory Issues (two subthemes), NIH (three subthemes), Journals (two subthemes), and Translation (two subthemes). After coding the data, text searches were conducted to bring together similar content and allow integration of the data through merging, a key methodological approach to integration in mixed methods study [23]. Qualitative quotations were used to illustrate and expand understanding of the diverse, sometimes concordant, and sometimes conflicting views. The instruments are fully described and available for download in our previous report limited to the ethical aspects of this part of the mixed methods evaluation of ADAPT-IT [15].

## Results

### Characteristics of participants

The survey was offered to 64 participants in the ADAPT-IT project, of whom 53 responded; in addition the survey was offered to 27 members of an NIH study section, of whom 23 responded (overall response rate 84 %). Overall, the median age was 49 years, the sample was split closely evenly between PhDs and MDs, and more than a third were women (Table 1).

**Table 1:** Characteristics of participants, *n* = 75. IQR represents range between 25^th^ and 75^th^ percentiles

Characteristic	Median	IQR
Age	49	44-56
Female, *n*, percentage	27	37 %
Highest degree		
MD or equivalent	43	57 %
PhD	32	43 %
Primary work location		
University/university hospital	47	64 %
Community hospital	5	7 %
Government (NIH or FDA)	15	21 %
Consulting firm	5	7 %
Other	2	3 %
Primary specialty (if physician)		
Neurology	23	55 %
Emergency medicine	9	21 %
Internal medicine	3	7 %
Other	7	17 %
Primary professional activity		
Clinical practice	10	13 %
Research	22	29 %
Teaching	6	8 %
Administration	13	17 %
Statistical	9	12 %
Government	17	22 %

### Overarching views about understanding and acceptability of ACTs in the trial community

**Fig. 1:**
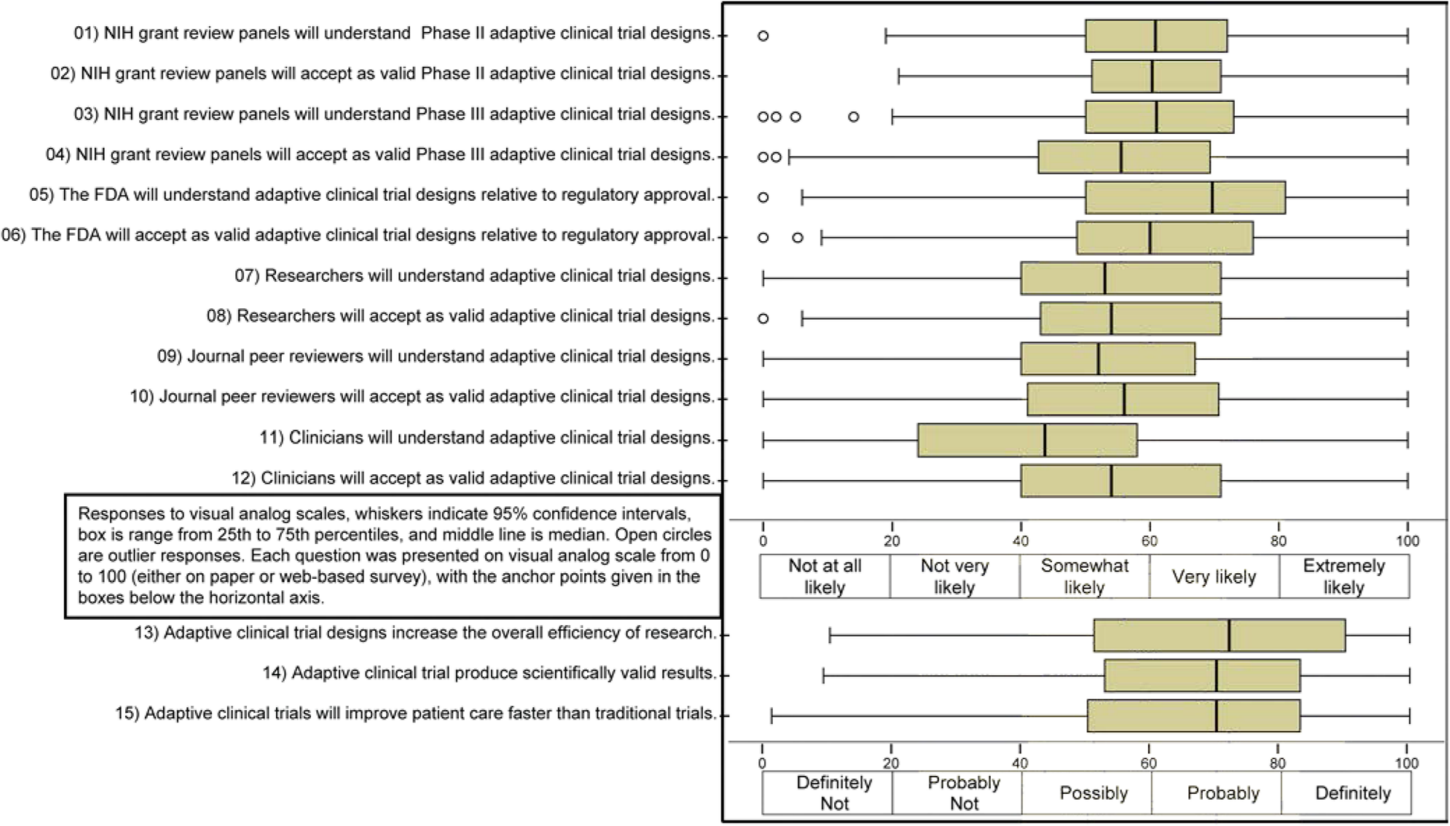
Distribution of attitudes about 15 aspects of ACTs for all respondents combined. *Center lines* represent median, *boxes* represent 25^th^ and 75^th^ percentiles, and *whiskers* represent the highest and lowest observed values that fall within 1.5 times the interquartile range

Respondents assessed understanding and acceptability of adaptive designs from the perspective of five major constituencies: the FDA, NIH review panels, researchers, clinicians, and journal peer reviewers. The overarching opinions are provided in Fig. 1. From the VAS data overall, the respondents were generally positive towards adaptive designs. Specifically, it appears that the respondents as a group felt that the FDA was most likely to understand adaptive designs and that clinicians were least likely to understand these designs.

From the focus group transcripts, we identified specific responses that seemed to exemplify the VAS findings. One clinician trialist’s general view about the entire funding implementation and publishing enterprise was that clinicians would accept adaptive design studies which were published in major peer-reviewed journals. However, she thought it unlikely that clinicians would understand them. In her opinion, review was a matter of luck. At study section the study is flagged as an adaptive design, and the “SRA (scientific review administrator) would find someone with expertise to review it.” This guaranteed “the reviewing statistician would understand the protocol, even if they did not like it.” For a journal, she felt that review was much more a “roll of the dice.”

To better understand how views of the clinical trial community vary by constituency and specific issues, we further examined respondents’ views more closely, constituent by constituent, as illustrated below.

#### Understanding of adaptive designs by NIH review panels

**Fig. 2:**
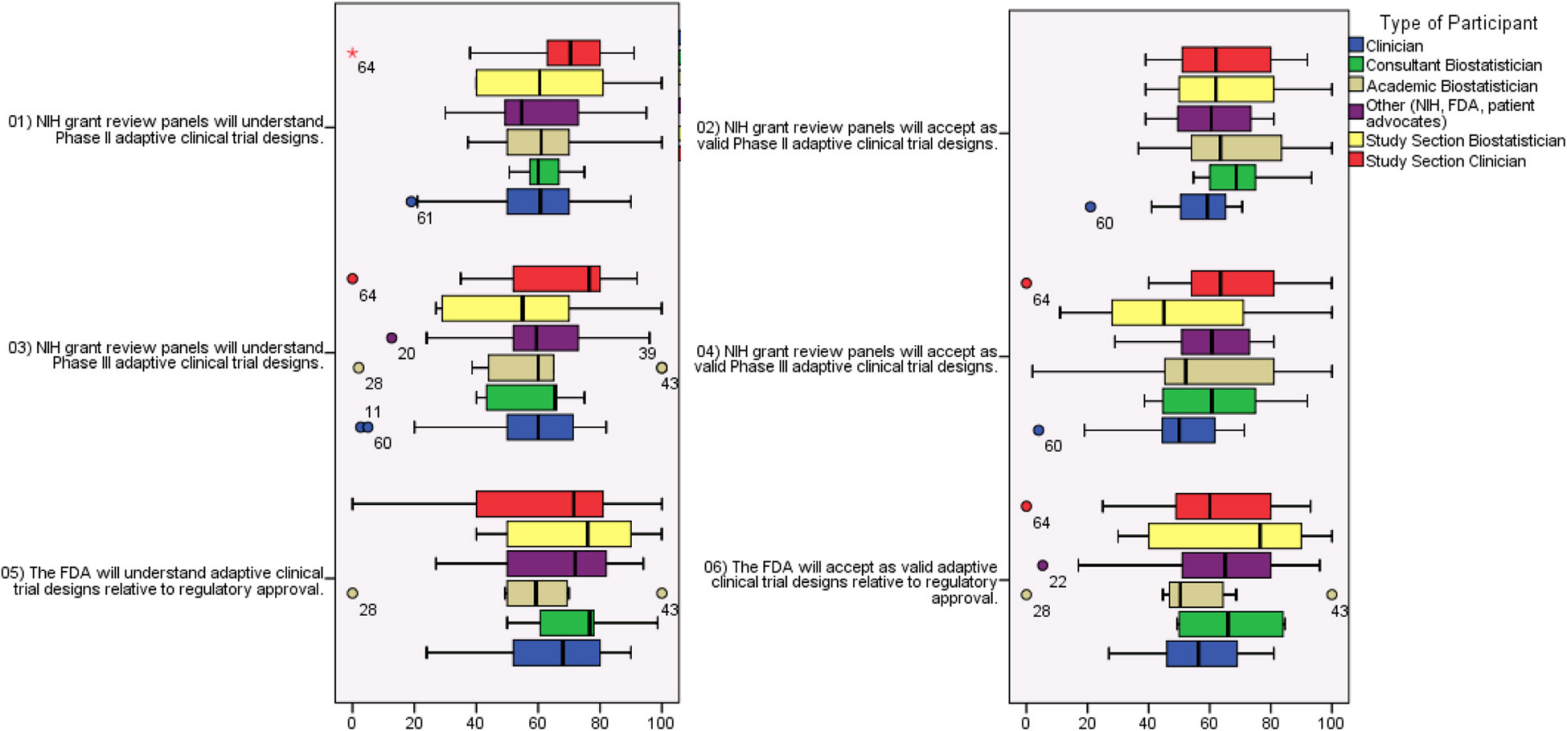
Perceived understanding and acceptance of adaptive designs by FDA and NIH. *Center lines* represent median, *boxes* represent 25^th^ and 75^th^ percentiles, and *whiskers* represent the highest and lowest observed values that fall within 1.5 times the interquartile range

The respondents generally had neutral to mildly positive attitudes regarding the extent to which NIH review panels would understand and accept adaptive design proposals (Fig. 2). Among all groups, the academic and study section biostatistician respondents had noticeably less optimistic views regarding the understanding of phase III adaptive trials by NIH study sections, particularly relative to phase II proposals. Widely dispersed VAS responses by these groups indicate varied opinions within this group with strongly polarized feelings that NIH peer review panels will accept or not accept phase III adaptive designs as valid. For example, a consultant statistician noted that many reviewers may not have been trained in or familiar with adaptive design methods. A clinician trialist echoed the belief that statisticians at the NIH review (committee) and clinical researchers who believe they understand statistical design on the NIH review (committee) will probably not understand the adaptive design and how it is applied to the phase II study.

Regarding process, a clinician trialist opined that the statisticians would become the default authority at study section. The trialist expressed the notion that statisticians on the panels would understand the adaptive design, while clinicians would not. Rather, the clinicians would ask the statisticians for their opinion and defer to their views. Others noted that even among statisticians there is a large range of experience and that there is limited coverage of adaptive designs and clinical trial simulation within most PhD biostatistics curricula. Pessimism about the ability to find qualified reviewers reinforced concern about ACTs getting a fair review. Respondents worry that NIH review panels may not have biostatisticians with experience in more complex adaptive designs. They also cite the short page limits and the challenge study sections have to understand the complex designs, given a reduced ability to reproduce the sample size estimations (whereas more fixed designs using standard frequentist *t* tests or differences in proportions are easy to replicate quickly). One clinician trialist summarized a common sentiment that the statistical expertise did not currently exist on NIH review to completely understand the adaptive design process, except in concept. Their hope was that the NIH would be willing to allow adaptive design studies to move forward if developed by well-recognized experts.

#### Acceptance of adaptive designs by NIH review panels

To compare with questioning about understanding, we also solicited opinions about the respondents’ perceptions of NIH review panel acceptance of adaptive designs. As with understanding, respondent opinions ranged from pessimistic to very optimistic regarding acceptance (Fig. 2). A consultant statistician optimistically stated that “forms of these designs have been used for many years” and, in their opinion, the designs were “demanded” by the NIH. In contrast, an academic biostatistician felt that NIH panels may be more likely to accept adaptive designs even if they do not necessarily understand them, mainly because they are considered to be innovative and provide other efficiencies. In the middle ground, a study section biostatistician felt that acceptance would depend on the situation and only after consideration of whether an adaptive design would confer an advantage. It was this biostatistician’s opinion that not all adaptations are the same, and some approaches may be valid for early phase trials but not for confirmatory trials. The idea of adaptation seems at odds with the notion of a “confirmatory” clinical trial, except for narrowly defined adaptations, e.g., blinded sample size re-estimation. For this reason, adaptive designs are valid, but not always better than traditional approaches. In the opinion of this biostatistician, like traditional trials, adaptive design trials need to be properly designed, executed, and analyzed.

Many respondents felt that even if there were inadequate understanding of adaptive designs, there would still be enthusiasm based on the novelty of designs. For example, an academic biostatistician expressed the belief that reviewers may be more likely to accept phase II designs as valid (over phase III) because they were not confirmatory trials. Further, the biostatistician thought that some reviewers may accept adaptive designs as valid, even if they did not completely understand the methodology. A clinician trialist felt that many clinicians will accept what statisticians tell them are valid designs. Another clinician trialist summarized his thoughts about understanding and acceptance, saying that while many clinicians will not understand these designs, the clinical trials study section (at least at the National Institute of Neurological Disorders and Stroke, NINDS) had statisticians who are very capable of understanding adaptive design trials, whether or not they accepted them as the best way to conduct a trial. The clinician felt that this was the same case for the FDA. Another participant expressed trepidation of the “safety” of submitting an adaptive design, and concerns that adaptive designs would not be submitted to the FDA. In their opinion, investigators in need of funding would hesitate to submit adaptive design analysis that might be perceived as too novel. Therefore, it was safer to design trials using standard statistical methods, even if they were not optimal.

### Understanding and acceptance: opinions regarding FDA review

While opinions varied greatly, in general the participants perceived that the FDA would have the greatest understanding of adaptive designs. As illustrated in Fig. 1, respondents generally rated the FDA as having a high likelihood of understanding ACTs. Interestingly, the same respondents overall were not as convinced that better understanding translates into acceptance of ACTs. This did not seem to vary significantly by constituency affiliation (Fig. 2), although academic biostatisticians appeared to have more neutral views regarding FDA understanding.

#### Understanding of adaptive designs by the FDA

Generally, participants believed that the FDA understands adaptive designs, since the FDA has issued guidance documents on adaptive design clinical trials. However, others believed there is more heterogeneity of understanding within the FDA. One academic biostatistician felt that the degree of understanding depended on the branch of the FDA and the specific group of statisticians. The reason for the faith in understanding by the FDA aligned with the perception that the FDA has the most previous experience with ACTs. An FDA scientist pointed out that the FDA has accepted well-understood adaptations for years, but some more difficult adaptive designs may be better understood than others.

#### Acceptance of adaptive designs by the FDA

Respondents also expressed some doubt as to whether the FDA’s better understanding of complicated adaptive designs would translate into the FDA accepting the results due to perceived problems with broader interpretability. According to a clinician trialist, the FDA would not accept any type of analysis that could leave open the possibility that the result occurred because of chance or unforeseen circumstances. This trialist felt that the FDA desires simple designs so that they can unequivocally support the primary outcome measure and so that practitioners can easily understand the results. Therefore, the FDA may understand the designs but may not accept the results as valid. This opinion contrasts with that of other respondents who more optimistically pointed out that the FDA has a major push towards improving the efficiency of the medical device approval process and that adaptive designs are to be a part of that. A consultant biostatistician noted the FDA’s history of acceptance of new methods, pointing out that the Critical Path Initiative encouraged the FDA to accept well-designed adaptive trials. In further contrast, others felt that under a broad definition of “adaptations,” most currently conducted trials are adaptive and are generally accepted as such. An academic biostatistician explained that:

*The NIH and FDA already accept adaptive designs based on pre-specified blinded analysis of interim results, as well as pre-specified group sequential designs. Other researchers have considered adaptive modification of randomization ratios across treatment arms or within strata defined by eligibility criteria. (Such can in some sense be viewed as including adaptive dropping of treatment arms or subgroups.) In my experience, the FDA has already accepted adaptive selection of subgroups and doses based on interim estimates of treatment effects when those rules were pre-specified in an easily understood manner.*

Clinicians expressed a need for the adaptive design to justify itself to the FDA (and NIH). A clinician trialist felt that a study would have “to be improved by an adaptive design, either by making it cheaper or easier to interpret, in order for NIH or FDA to prefer that design over the frequentist approach.”

### Understanding and acceptance: respondent opinions regarding the clinical and research community

**Fig. 3:**
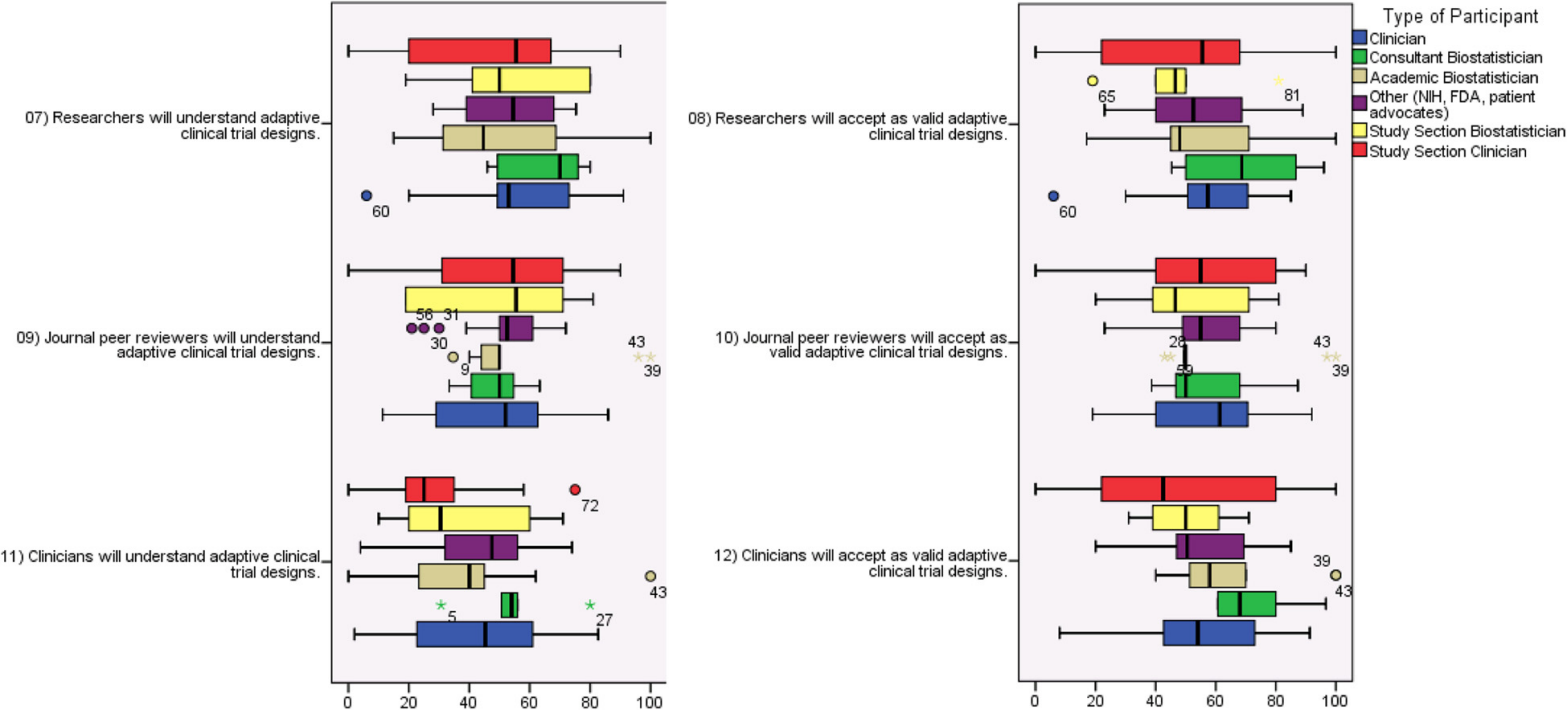
Perceived understandibility and acceptability of adaptive designs by researchers, clinicians, and journal peer reviewers. *Center lines* represent median, *boxes* represent 25^th^ and 75^th^ percentiles, and *whiskers* represent the highest and lowest observed values that fall within 1.5 times the interquartile range. Variability perceived by clinicians and study section clinicians. (Consultants have journal peer reviewer bad experiences.)

Adaptive designs were felt to be understood variably across the constituencies of researchers, clinicians, and journal peer reviewers (Fig. 3). Moreover, respondents generally believe that clinicians and clinician researchers have limited understanding of ACTs. The sources of the diverse views about the ability to understand and to accept adaptive designs are expressed through the qualitatively collected comments. In contrast to the extensive experience in the private world, some respondents noted the impact of limited experience in the academic setting as an impediment to understanding adaptive designs. An academic biostatistician noted that although adaptive design trials are becoming more common, several researchers, particularly in the academic setting, have little experience designing and conducting phase II adaptive designs. More experience would allow for better understanding of these designs. The respondents expressed concern that when research results are published, the broader medical community would have very little understanding of the actual design. A patient advocate felt that researchers and peer reviewers would not understand or accept what has been provided, unless there was legitimate and applicable proof. Further, they felt that clinicians were more apt to understand and accept new clinical trial design approaches. In addition, a FDA scientist expressed the concern that clinicians would not understand the results from ACTs and felt this was an important area that needed more discussion.

### Understanding and acceptance: opinions regarding the statistical community

While the respondents were not asked to specifically opine regarding statistician attitudes towards adaptive designs in the VAS instruments, focus group discussions often involved opinions regarding how statisticians from various venues including the FDA, academia, and industry would be expected to respond to adaptive designs. Opinions regarding statistical experience mirrored what was thought of the broader academic research community in clinical trials: Understanding is highly variable, and the majority of statisticians in academic practice have limited experience with more complex adaptive designs. Respondents pointed out that statisticians have variable experience with these designs, ranging from nearly no experience to the design of hundreds of adaptive trials. Other barriers that the biostatistician mentioned were the lack of print exposure and training, noting that often statisticians are not given much room to describe the design in detail in a clinical journal. Preconceived biases on the part of statistical reviewers were an additional hindrance.

The respondents also noted that clinical researchers generally expect all statisticians to be experts in all types of clinical trial designs, despite the large range of experience observed among statisticians and the limited coverage of adaptive designs and clinical trial simulation within most PhD biostatistics curricula. Another view among some respondents is that varied understanding among different constituencies should be expected, but that designs should not circumvent good science. An academic biostatistician noted that generally the important operating characteristics between adaptive and non-adaptive designs are not radically different. This biostatistician also stated that strong advocates of adaptive design trials should emphasize the ways that adaptive designs could be used to strengthen the scientific method.

### Opinions regarding efficiency, validity, and potential to speed discovery

**Fig. 4:**
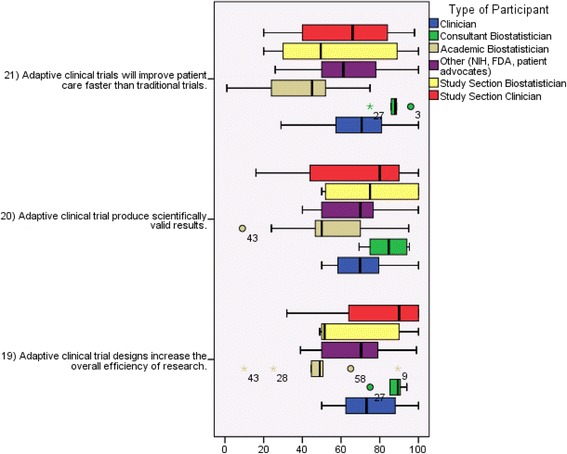
Perceived efficiency and scientific validity of adaptive designs. *Center lines* represent median*, boxes* represent 25^th^ and 75^th^ percentiles, and *whiskers* represent the highest and lowest observed values that fall within 1.5 times the interquartile range

In addition to the comparisons of respondents’ views about understanding and acceptability in the research community, we also examined the domains of efficiency, scientific validity, and speed of discovery. Figures 1 and 4 illustrate respondents’ attitudes about efficiency, scientific validity, and speed of discovery. In general, the respondents were optimistic about the potential for ACTs on these subjects, particularly on the efficiency domain. In addition, academic biostatisticians provided responses in the neutral range on these three domains. The textual responses below illustrate some important nuances to this optimism.

### Efficiency may be increased, decreased, or unchanged by adaptive designs depending on the planning and implementation

The respondents provided several views on efficiency from the mini-focus groups. One example is that it seems that clinicians/non-statistician researchers generally believe that adaptive designs can simply reduce the overall sample size. This efficiency was an advantage that would offset the perception that the analysis or adaptive designs were convoluted and hard to follow. An academic biostatistician pointed out that adaptive designs may or may not reduce the sample size, but they will generally change the number of scientific questions that can be answered quantitatively and offer other advantages. The sample size was dependent on the phase of the trial, what was being adapted, and how the researchers defined efficiency. In the opinion of an academic biostatistician, a comparison of a trial completed under different designs was needed to answer the question of what advantages adaptive design trials conferred.

In another twist, some have strong opinions regarding the potential for increased efficiency. A consultant biostatistician stated that adaptive design trials are more efficient in terms of patients and resources, saying that there could be huge savings in running a clinical trial. They further opined that the “non-adaptive” approach was leading the USA into bankruptcy due to health care costs. A consultant biostatistician expanded further:

*Efficiency may be conceptualized either as identifying effective treatments from a large pool of candidates or as minimizing the “cost” of answering a discrete clinical question. The opportunity cost of conducting any trial (in terms of not being able to dedicate resources to answering other questions or exploring other treatments) is not frequently a design or review consideration. While adaptive designs may improve the efficiency of finding treatments from a pool of candidates, the benefits within a single constrained question may be more limited.*

An academic biostatistician pointed out that adaptive trials could increase the number of questions at each stage of a trial “at the price of having less information with which to address some of these questions.” One academic biostatistician lamented that some adaptive designs tended to decrease the overall efficiency of research due to the lack of attention to the adaptive rules that had been chosen.

A clinician trialist pointed out that adaptive designs produce valid results; however, if primary outcomes are different enough, researchers may lose the power with which to study secondary outcomes (for example, if too few patients are assigned to an arm that “loses” on primary outcome). This could be detrimental, since there are times when the secondary outcomes are also important. In an example trial, two antiepileptic drugs (AEDs) were compared head to head. The clinician trialist describes this scenario in further detail: “Drug A is found to be more effective but patients develop many more cognitive side effects. In a traditional trial design Drug A ‘wins’ on primary outcome (efficacy) and ‘loses’ on secondary. In an adaptive design Drug A would win on cognitive function but may have insufficient power to determine whether secondary outcomes were significantly worse.”

### The validity of inference derived from more complicated ACTs is an area of varied opinions

Most groups indicated overall optimism regarding validity; however, the academic biostatisticians appear to have a more neutral view (Fig. 4). The textual responses show that clinicians and non-statistician researchers may not be able to assess the validity of ACT designs and will generally defer to their known biostatistician collaborators, who may or may not actually have experience with the designs in question. An academic biostatistician explained that in his experience clinicians embraced adaptive designs; however, they were in danger of accepting their validity without understanding the statistical details. According to an academic biostatistician, the key to the scientific method is the isolation of questions that an experiment is designed to answer. They feared adaptive designs can be used improperly, blurring the definition of the scientific hypothesis that is answered. Therefore, proper interpretation of the results must guard against the possible bias of investigators in reporting results that may diminish the true gains that are possible with a systematic adaptive approach to addressing many alternative hypotheses. Their recommendation was that the scientific community should understand the multiple hypotheses that were a priori under consideration, in order to understand and interpret the adaptive randomized clinical trial results.

As direct comparative study between more complicated designs alongside more traditional, simple designs is lacking and mostly limited to simulation studies, respondents argued the need for more experience with adaptive designs. According to a patient advocate, as experience grows and ACT designs are used more universally, efficiency in clinical research should improve. Errors and misunderstandings would be minimized with greater experience, and the improved reliability of results would result in great acceptance by clinicians and patients.

### The structures and traditions of the clinical trial process are relevant to the consideration of adaptive design in the “learning” and “confirming” phases

A clinician trialist noted that phase II trials have typically been focused and smaller. This trialist noted that many assumptions regarding inclusion population, dose, and duration of treatment are made and cemented here. He also noted that the opportunity to make many of these decisions prospectively and quantitatively is lost, and instead the phase III design may be informed by underpowered subgroup analyses from these smaller trials.

As a clinician trialist pointed out, phase II trials using adaptive designs may be larger than phase II trials in the past; however, this enables a smaller, more focused phase III study with better controls for type I and type II errors. This trialist noted that the idea of inversion (larger phase II and smaller phase III trials) would run counter to long traditions at NIH and in academia.

An academic biostatistician had detailed thoughts regarding the current atmosphere of clinical trials and how decisions to proceed from phase II to III are conducted.

*I hope the NIH, in particular, will adopt phase II strategies that use adaptive design strategies to better control the type I and type II errors as treatment investigations proceed from phase II to phase III. As a general rule, the setting and conditions under which phase III studies are conducted do not exactly mirror those of the antecedent phase II studies, in part due to a certain amount of “data-dredging” of the phase II data…..Were the NIH to take a more systematic approach to evaluating the phase II to phase III transition, the prevalence of “negative” phase III studies could be greatly decreased.*

## Discussion

### The respondents had varying opinions regarding adaptive trials

We observed diverse opinions regarding ACT designs. We provided no definition of adaptive designs or framing to the participants prior to their completing the VAS surveys other than that this was an FDA/NIH project on adaptive designs for confirmatory trials. Most responses to VAS surveys were in a neutral range; however, some differences across constituency were observed. Participants generally believed that clinicians in practice will have the most difficulty understanding ACT designs, whereas the FDA will understand such designs relative to regulatory approval. This suggests the need for greater education of the broader community regarding ACT designs. Responses from study section members were more varied. Overall the study section respondents indicated that they believed NIH review panels would be able to understand phase II and III trials and be able to accept phase II trials more than phase III trials. Interestingly, study sections seemed to have a lower opinion regarding the potential for ACTs to improve patient care faster than traditional trials.

### Important implications of this work for each constituency studied

The current investigation has several important implications for the various constituencies we studied. We consider the academic paradigm of clinicians conceiving of a trial design, collaborating with biostatisticians to refine the scientific experiment, submitting to the NIH for a funding decision (with peer review by clinicians and biostatisticians), and regulators (FDA) making decisions about approving treatments for use. For clinicians, the implications of our observations are clear: Better understanding is needed so that current enthusiasm for adaptive designs can be harnessed in a scientifically useful way. An important further area of work is improved insight that allows clinicians to understand when adding complexity can be helpful and worth the cost. The most important implications for biostatisticians involve communication, both among themselves and to the clinical audiences. Our findings are distinct from the industry-focused surveys of the Adaptive Designs working group, where it seems that communication is perceived as a lessening barrier [11]. Adaptive designs require more upfront planning, and the communication of simulations is quite difficult, even with an in-person meeting. The communication of a complicated design within a short grant application is a big challenge. Our work’s important implication for funders also stems from these communication and development issues: Mechanisms should be put into place to allow for the careful simulation and description of designs before funding, and possibly even for “design competitions” prior to submitting full trial proposals. This clearly would introduce major challenges (reference scenarios to simulate), but given the current low success rate for phase III clinical trials, it is a change that would likely accelerate scientific discovery [24–27]. In addition, a rethinking of the “small” learning phase II trial (where little is learned) and the larger phase III trial (where assumptions made on limited data from the smaller phase II trial are enforced as fixed trial characteristics) may be worthwhile for governmental funders to consider [28, 29]. Finally, the implications for regulatory bodies of this work are interesting. In general, regulatory agencies are deemed to have the most experience and scientific knowledge of the performance of these designs, although they are frequently believed to be conservative. Methods to capitalize on this expertise (serving on NIH review panels and helping to set up “design competitions”) across governmental trial development initiatives should actively be sought.

### Scientific dissatisfaction with the current clinical trial development pathway is common

The qualitative analysis provided important insights into the domains of acceptability, understandability, validity, and the potential efficiency of ACT designs. In addition, several responses addressed an important limitation in the current trial development process within the NIH: Phase II trials typically do not lend themselves to quantitative decision making regarding the most promising agents, treatment regimens, and populations to investigate further in phase III trials. Clinician trialists, academic biostatisticians, consultant biostatisticians, and others highlighted this major current limitation in the traditional approach to clinical trials and perceived major barriers in many constituencies to “inverting” the discovery progress to have larger, more informative phase II trials, followed by more focused and likely smaller phase III trials. The current approach, which involves a currently high prevalence of phase III trials whose results do not well inform clinical practice, was described unfavorably. One respondent related that the “data-dredging” that often goes on between phase II and III is contributing to failure by the pursuit of false signals, and that a more coordinated, quantitative approach with pre-specified decision rules could improve the impact of phase III trials.

### Adaptive designs have more to offer than reduced sample sizes, yet it is difficult to quantify the benefit of flexibility

Next, an important area of response is the potential efficiency gained from the incorporation of adaptive designs. Many of the clinician trialists participating had little background with adaptive trials at the time of data collection. As such, the prevailing opinion was that adaptive designs offer great promise because they can reduce sample size and thus the cost of the study. Some clinicians did have some experience and had unique insights. One response in particular, regarding the tradeoff between learning about the efficacy of several AEDs versus more precision in learning about the side effects of the agents with precision, was telling. Regardless of ultimate design decisions for adaptive versus more traditional designs, open discussion of the value of the diverse sets of clinical data collected during a trial is clearly important. With respect to efficiency, biostatisticians and regulatory scientists had a broader view. While it is possible to reduce sample size, answer more questions, or terminate trials of non-efficacious treatments sooner in many cases, great care must be taken to ensure that good scientific principles are followed in experimental design. Some previous adaptive designs that these constituents have experienced may have been less rigorous; therefore, many responses discussed the great diversity in what sorts of designs are truly adaptive.

### A lack of a common language inhibits the uptake and use of adaptive trials

Building upon the diversity of clinical trials considered adaptive, the definition of an ACT was elusive and varied among the constituents in this baseline mixed methods study. Many of the consultant statisticians who had designed and conducted a large number of trials felt that the pre-specification and simulation of the entire design was implied in the term: adaptive clinical trial. On the other hand, academic biostatisticians, clinician trialists, and study section members had a less specific view and broadly considered the terminology to include trials with ad hoc changes based on accumulating data and relatively well-established group sequential designs. As a result, in many responses these groups were describing different types of adaptive designs. It was pointed out that “adaptive clinical trials” have become desirable, and that the term has become a buzzword of sorts. Other work has previously attempted to provide a taxonomy for describing adaptive designs [30], and the FDA has provided guidance on “generally well-understood” adaptive designs and also classified some types of designs as needing further research and validation [3]. Overall, this lack of an intuitive common language represents a large current barrier as clinicians and biostatisticians work to design clinical trials. In addition, the scientific community needs specific efforts to improve the communication of these designs both among biostatisticians and to clinical audiences. Our work, which focused on interviewing principal investigators and statisticians developing clinical trial designs within the ADAPT-IT project, reinforced the need for more education and collaboration in academic trial development, particularly for more complicated adaptive designs.

### Limitations

This study has several important limitations. First, this was a relatively small study — however, key decision makers and clinical trialists were approached. The current study elicited opinions regarding the performance of ACT designs in various settings (NIH and FDA review, trial operation, knowledge translation), and these opinions may not reflect the reality regarding the response to ACTs in the broader biomedical community. While the ADAPT-IT project involved education, collaboration, and a shared design process among stakeholders from academia, industry, and government, this cross-sectional study occurred prior to any of the participants engaging in these activities together. It is likely that opinions, knowledge, and attitudes have changed after participating in ADAPT-IT project activities. Finally, we did not define ACTs for the participants prior to their completion of the VAS or interviews. Certain individuals with some experience with adaptive designs (particularly biostatisticians) may have felt unable to respond outside the neutral range without knowing what sort of adaptive designs the questions were referencing. This may be explained by a general ambivalence to adaptive designs within our stakeholders or by the great diversity of clinical trials that can be described as adaptive. In retrospect, our results may have changed if some additional information regarding the types of adaptive designs for which we wanted opinions were more explicitly stated. On the other hand, given that each individual brought his or her own internal definition to the table, we were able to collect rich textual comments that describe some of the potential benefits and drawbacks of several approaches.

## Conclusions

In summary, we found highly diverse opinions about the utility, efficiency, and stakeholder-specific understanding and acceptance of adaptive clinical trials. First and foremost, the definition of adaptive clinical trial varied across groups — when working in this area it is important to ensure that the types of adaptations under discussion are well described and understood. Next, clinician and non-statistician researcher understanding in this area is limited, and additional education and experience with the conduct and dissemination of adaptive designs seems the most important way to improve this. Similarly, statisticians have varied experience with adaptations. Nearly all biostatisticians involved with clinical trials have experience with group sequential designs, which could be considered the best-understood type of adaptive design. Far fewer have much experience with newer, more flexible and innovative types of adaptations. Important barriers to acceptance were identified, especially variable knowledge and potential misperceptions regarding how more innovative designs will fare in peer review. Many of the discussions highlight the problem with the structure of progression from phase II to III, an area that is widely recognized to be performing poorly in identifying treatments that prove to be successful in confirmatory trials. Since the phase II to III transition is generally not conducted according to pre-defined rules and data, this represents a potential area for improvement: namely, by conducting phase II trials that are better at selecting agents, doses, treatment regimens, and populations and facilitating objective, quantitative decision making about the structure of phase III trials. Finally, given the diversity of clinical trial designs considered adaptive, it is crucially important that the fundamentals of a sound scientific approach and progression be considered from the start of trial planning.

## Abbreviations

ADAPT-IT, Adaptive Designs Accelerating Promising Trials into Treatments; ACT, adaptive clinical trial; NIH, National Institutes of Health; NINDS, National Institute of Neurological Disorders and Stroke; FDA, Food and Drug Administration; NETT, Neurological Emergencies Treatment Trials; LLC, limited liability company; VAS, visual analog scale

## Additional file

Additional file 1: ADAPT-IT general-coreq-FINAL. Consolidated criteria for reporting qualitative research (COREQ) checklist contents: responses to the 32-item COREQ checklist as applicable to the current research study. (DOCX 26 kb)
