# Supplementary material for: Attitudes and opinions regarding confirmatory adaptive clinical trials: a mixed methods analysis from the Adaptive Designs Accelerating Promising Trials into Treatments (ADAPT-IT) project
Source: Trials. 2016 Jul 29;17:373. doi: 10.1186/s13063-016-1493-z (PMC4966769; doi:10.1186/s13063-016-1493-z)
Supplement: Additional file 1: — ADAPT-IT general-coreq-FINAL. Consolidated criteria for reporting qualitative research (COREQ) checklist contents: responses to the 32-item COREQ checklist as applicable to the current research study. (DOCX 26 kb) [file 13063_2016_1493_MOESM1_ESM.docx]

**Domain 1: Research team and reflexivity**

***Personal Characteristics***

1.Interviewer/facilitator: Which author/s conducted the interview or focus group?

*Drs. Legocki and Fetters conducted the mini-focus groups.*

2.Credentials: What were the researcher's credentials? E.g. PhD, MD

Dr. Legocki has a PhD and Dr. Fetters an MD, MPH, and MA.

3.Occupation: What was their occupation at the time of the study?

*Dr. Legocki was a research investigator and Dr. Fetters was a professor in the Department of Family Medicine at the time of this study.*

4.Gender: Was the researcher male or female?

*Dr. Legocki is female; Dr. Fetters is male.*

5.Experience and training: What experience or training did the researcher have?

*Both Drs. Legocki and Fetters have extensive experience in qualitative data collection and analysis. Both are experienced mixed-methods researchers and have designed and conducted numerous qualitative and mixed-methods studies. In addition, Dr. Fetters has provided multiple international workshops on qualitative and mixed methods research.*

***Relationship with participants***

6.Relationship established: Was a relationship established prior to study commencement?

*As part of the mixed methods evaluation team, neither Dr. Fetters or Legocki had previous involvement with the statistics teams or clinical researchers designing these trials.*

7.Participant knowledge of the interviewer: What did the participants know about the researcher? e.g. personal goals, reasons for doing the research

As part of the consent process Drs. Legocki and Fetters described the purpose of the study, their role and experience.

8.Interviewer characteristics: What characteristics were reported about the interviewer/facilitator? *e.g.*Bias, assumptions, reasons and interests in the research topic

Drs. Legocki and Fetters were recruited by the researchers from the Neurological Emergencies Trials Treatment (NETT) Network (Dr. Barsan is the Principal Investigator of the Clinical Coordinating center), to conduct the evaluation portion of the ADAPT-IT project. Drs. Fetters and Legocki had no prior experience or pre-conceived notions regarding adaptive clinical trials. They told subjects that their interest was in learning more about the participants views about adaptive clinical trials.

**Domain 2: study design**

***Theoretical framework***

9.Methodological orientation and Theory : What methodological orientation was stated to underpin the study? e.g. grounded theory, discourse analysis, ethnography, phenomenology, content analysis

The researchers did not tell the subjects that they were using a specific methodological orientation. However, the methodological orientation was ethnography as the senior researcher, Dr. Fetters, trained in medical anthropology for his MA degree. The researchers described themes that emerged from the mini-focus groups and textual responses to the surveys.

***Participant selection***

10.Sampling: How were participants selected? e.g. purposive, convenience, consecutive, snowball

The sampling strategy was purposive, specifically, maximum variation sampling. Clinicians, biostatisticians and others who were to be involved in the planning of clinical trials within this project were selected to participate in the VAS questionaires and mini-focus groups. This selection process occurred as part of the initial grant submission (to identify principal investigators designing trials, along with clinical and statistical representatives of the NETT network, along with the private statistical consulting group with adaptive clinical trial design experience.) We also were given a list of emails of prior members of an National Institutes of Neurological Disorders and Stroke (NINDS) clinical trials study section. Data collection from this group was limited to anonymous surveys.

11.Method of approach: How were participants approached? e.g. face-to-face, telephone, mail, email

Both face-to-face and email strategies were used at different points in the study. Initially, participants were approached face-to-face as part of the main grant proposal development. After funding, we recruited project team members by email using contacts with each network along with clinicians and statisticians who worked with the PIs for each trial that was being planned. For the study section members, an initial email and one reminder invited them to the web only VAS survey with space for text responses.

12.Sample size: How many participants were in the study?

*A total of 76.*

13. Non-participation: How many people refused to participate or dropped out? Reasons?

*See results section. 64 ADAPT-IT participants and 27 study section members received the survey. All individuals invited to the mini-focus groups attended. This was a subset of the overall VAS response population. Eleven subjects were distributed across 3 mini-focus groups.*

*Setting*

14. Setting of data collection: Where was the data collected? e.g. home, clinic, workplace

VAS data was collected either as part of a paper survey immediately before an in-person Adapt IT project development meeting at a hotel or via an online survey. Mini-focus groups were conducted in hotel conference rooms immediately before the first set of trial planning meetings.

15. Presence of non-participants: Was anyone else present besides the participants and researchers?

*No*

16. Description of sample: What are the important characteristics of the sample? e.g. demographic data, date

The demographics section of the manuscript results contains relevant details of the participant demographics. There are sufficient details to understand particpants’ backgrounds in aggregate, but insufficient information to identify individuals personally

***Data collection***

17. Interview guide: Were questions, prompts, guides provided by the authors? Was it pilot tested?

*Both the VAS survey and the discussion guide were pilot-tested on emergency medicine faculty members, otherwise completely un-involved with the ADAPT-IT project. These faculty had experience in clinical trial design and experience conducting focus groups of health care providers.*

18. Repeat interviews: Were repeat interviews carried out? If yes, how many?

*As part of baseline data collection, no. A separate end of project study interview occurred but dealt with reflections on the trial planning process.*

19. Audio/visual recording: Did the research use audio or visual recording to collect the data?

*Audio recording and field observations made by Drs. Fetters and Legocki were used to collect the data. The audio recordings were used to create verbatim transcripts for analysis.*

20. Field notes: Were field notes made during and/or after the interview or focus group?

*As note above, field observations were taken by Dr. Fetters and Legocki using a structured format focused on context, content, and contextual ideas.*

21. Duration: What was the duration of the interviews or focus group?

*The mini-focus groups lasted approximately one hour.*

22. Data saturation: Was data saturation discussed?

*Data saturation was discussed and achieved. After review of the initial transcripts, and the results of the visual analog surveys data saturation was achieved based by consensus review of materials by the mixed methods team. In addition, given personnel overlap there was not felt to be additional benefit of conducting mini-focus groups prior to the introductory trial planning meetings of trials 3-5. Baseline VAS surveys were distributed to all unique participants at the beginning of each trial planning group.*

23. Transcripts returned: Were transcripts returned to participants for comment and/or correction?

*No. The initial transcripts received were reviewed by listening to the audio files. Corrections were made to accurately reflect the content.*

**Domain 3: analysis and findings**

***Data analysis***

24.Number of data coders: How many data coders coded the data?

*Dr. Fetters worked with two primary coders, Dr. Legocki and Ms. Frederiksen, to develop the coding scheme, and to develop calibration of the codes. Codes deemed challenging to apply or interpret, were discussed iteratively on an as needed basis.*

25.Description of the coding tree: Did authors provide a description of the coding tree?

*Provided below.*

26.Derivation of themes: Were themes identified in advance or derived from the data?

*We pre-specified a priori that some unique ethical issues would exist, along with issues of validity and efficiency. Additional themes were derived de novo from the data.*

27.Software: What software, if applicable, was used to manage the data?

*Atlas TI.*

28.Participant checking: Did participants provide feedback on the findings?

*No.*

***Reporting***

29.Quotations presented: Were participant quotations presented to illustrate the themes / findings? Was each quotation identified? e.g. participant number

Yes, quotations were presented to illustrate themes/findings. Participants were identified by role.

30.Data and findings consistent: Was there consistency between the data presented and the findings?

*We utilized a mixed methods research approach. The scaled data illustrate the range of scores from participants about various aspects of the study that the investigators chose a priori. The qualitative data were collected and presented thematically to the illustrate the breadth and variations in the quantitative findings.*

31.Clarity of major themes: Were major themes clearly presented in the findings?

*The major themes were used as a platform for the presentation of the study findings. There were intentionally linked with the quantitative visual analog scale findings.*

32.Clarity of minor themes: Is there a description of diverse cases or discussion of minor themes?

*The diverse cases and minor theme variations were discussed with the major themes.*

**Coding Tree**

| CODE | DEFINITION, INCLUSIONS, EXCLUSIONS, AND NOTES | ABBRIVIATION |
| --- | --- | --- |
| ACTs-General Issues | ACT relevant issues not addressed by other code below. ALWAYS use more specific code below if applicable. | ACT Gen |
| -experience | Describes individuals previous experiences with ACTs, including what went well, what could be improved. | ACT Exp |
| -impression | Describes general impression of ACTs including “definitions. Describes individuals view and opinions on what an adaptive trial is, but excludes comments about ambiguity | ACT Imp |
| -ambiguity | Describes sense of ambiguity about what constitutes ACTs including, what is adapted and at what stage of the trial are things adapted. Should be some implicit or explicit sense about confusion. | ACT Amb |
| -Reproducibility | Describes the reproducibility of ACTs | ACT Repro |
| Logistics | Logistical issues not addressed by other code below. ALWAYS use more specific code below if applicable. | Log |
| -designing/planning | Describes the logistics of designing an ACT through simulation. Also considers issues of sample size and significance. What is being adapted and when. | Log Design |
| -budgeting | Describes the logistics of budgeting for the implementation of an ACT. Contrast with fundability above. | Log Budg |
| -implementing | Describes the logistics of implementing this type of trial for the research team and the clinicians. | Log Implem |
| Ethics | Ethical issues not addressed by other code below. ALWAYS use more specific code below if applicable. | Eth |
| -patient perspective | Describes the advantages and disadvantages of ACTs for the patient including greater chances of receiving the drug that is doing better . Excludes comments on informed consent. | Eth Pat Persp |
| -researcher perspective | Describes the advantages and disadvantages of ACTs for the researcher such as ending a trial that is not working, switching participants from one arm to another. Gist of comment focuses on the perspective of the researcher. Contrast with Informed consent perspective researcher below. | Eth Researcher Persp |
| -societal perspective | Describes advantages and disadvantages, to society in general including the collective ethic or common good. Includes epidemiological and population perspectives. | Eth Society Presp |
| -Informed consent-patient perspective | Describes the challenges/issues of the **PROCESS** of informed consent from the patient perspective for conducting an ACT. | Eth Con Pat Persp |
| -Informed consent- physician/researcher perspective | Describes the challenges/issues of the **PROCESS** of informed consent from the physician/researcher perspective for conducting an ACT. Includes how to present information to patients. Gist of comment relates to the impact on the treating physician. | Eth Con Research Persp |
| Regulatory Issues | Regulatory issues not addressed by other code below. ALWAYS use more specific code below if applicable. | Reg Issue |
| -FDA Regulatory review | Includes comments regarding how the FDA responds(ed) to adaptive trial designs from a regulatory perspective; phase II and III, basic understanding and resistance. Includes concerns regarding validity issues within the FDA, will they view the results of ACTs as valid | Reg FDA Review |
| -IRB | Describes the challenges/issues of working with IRBs relative to conduct of ACTs | Reg IRB |
| NIH | NIH relevant issues not addressed by other code below. ALWAYS use more specific code below if applicable. | NIH |
| -peer review | Describes how have NIH grant review panels respond(ed) to adaptive trial designs; both phase II and III. | NIH Peer review |
| -fundability | Describes fundability of ACTs from NIH perspective. Excludes “up-front” costs. | NIH Fund |
| -logistics of NIH grants | Describes logistical issues with NIH grants including page length of proposals, ACTs as innovative idea for funding, adaptive as a buzz word for funding. | NIH Grant log |
| Journals | Journal relevant issues not addressed by other code below. ALWAYS use more specific code below if applicable. | Journ |
| -pre-publication | Describes issues of those reviewing manuscripts including basic understand and ability to accurately evaluate ACT trials, including availability of people qualified to serve as reviewers. Will editors accept these types of articles, be willing to publish them | Journ Pre-pub |
| -Post-publication | Describes thoughts on how ACTs will be received among the readership of clinical journals. Includes how ACTs will be viewed and understood by clinicians, specifically will they see results from ACTs as valid and implement them into practice. 4/28/11 | Journ Post-pub |
| Translation | Translation relevant issues not addressed by other code below. ALWAYS use more specific code below if applicable. | Tran |
| Clinical | Clinical acceptability, translation into clinical practice | Tran Clin |
| Community | Community/public acceptability | Tran Commun |
